# Rethinking Urban and Food Policies to Improve Citizens Safety After COVID-19 Pandemic

**DOI:** 10.3389/fnut.2020.569542

**Published:** 2020-10-08

**Authors:** Andrea Galimberti, Hellas Cena, Luca Campone, Emanuele Ferri, Mario Dell'Agli, Enrico Sangiovanni, Michael Belingheri, Michele Augusto Riva, Maurizio Casiraghi, Massimo Labra

**Affiliations:** ^1^Department of Biotechnology and Biosciences, University of Milano - Bicocca, Milan, Italy; ^2^Laboratory of Dietetics and Clinical Nutrition, Department of Public Health, Experimental and Forensic Medicine, University of Pavia, Pavia, Italy; ^3^Clinical Nutrition and Dietetics Service, Unit of Internal Medicine and Endocrinology, Istituti Clinici Scientifici Maugeri Istituti di Ricovero e Cura a Carattere Scientifico, Pavia, Italy; ^4^Department of Pharmacological and Biomolecular Sciences, University of Milan, Milan, Italy; ^5^School of Medicine and Surgery, University of Milano - Bicocca, Monza, Italy

**Keywords:** virus, spillover, food safety, micronutrients, post-normal science

## Abstract

The ongoing pandemic caused by the coronavirus disease 2019 (COVID-19) is literally changing the world. From December 2019 to date, more than 22 million cases have been reported worldwide and global health institutions are acting to slow down the virus transmission and are looking for possible prevention strategies in case of a new outbreak. As in other endemic or pandemic phenomena, the issues mostly covered by scientific and media attention are related to the diagnostic and therapeutic approach of COVID-19. However, a still neglected issue regards the adoption of a more systemic approach considering the close connection among the infection, the environment, and human behaviors, including the role of diet and urban management. To shed light on this issue, we brought together a faculty group involving experts in environment and biodiversity, food safety, human nutrition, and behavior, bioprospecting, as well as medical doctors having a deep knowledge of the complex historical relationship between humanity and vector-borne infections. Two main aspects emerged from the integrative overview of the current COVID-19 pandemic: (i) the scientific community should start sharing social actions and policy advocacy based on the assumption that human health strongly depends upon a sustainable exploitation of natural resources in populated areas; (ii) the specific strategic role of the cities in developing sustainable food systems and promoting healthy dietary patterns. Definitely, some priority issues should be addressed to achieve these goals, such as global efforts to increase food safety and security, which would benefit from urban and peri-urban agriculture enhancement, smallholder food producers support, and ecosystem services and local biodiversity maintenance.

## Introduction

The ongoing pandemic caused by the coronavirus disease 2019 (COVID-19) is literally changing the world (1, 2). From the first documented human patient in Wuhan (Hubei, People's Republic of China) in December 2019, on August 2020, more than 22 million cases have been reported worldwide, of which more than six million still active (1% in serious or critical conditions) and almost 800 k deaths. WHO and other authorities soon realized that it was no longer possible to contain the virus spread, but only to slow down its transmission and try, at least, to reduce “pressure” on national health systems. As in other endemic or pandemic outbreaks, the issues mostly covered by scientific and media attention are related to the diagnostic and therapeutic approach of COVID-19 contagion. However, greater consideration should be given to a systemic approach considering the close connection between this disease, the environment and human behaviors, in a framework of building a safer, more sustainable and healthier world (3). How is it possible that a virus from a Chinese market has spread to other continents so quickly, penetrating the heart of cities and killing the weakest citizens?

To shed light on this issue, we brought together a faculty group involving experts in environment and biodiversity, food safety, human nutrition, and behavior, biological activity of natural products as well as medical doctors having a deep knowledge of the complex historical relationship between humanity and vector-borne infections.

**Figure 1:**
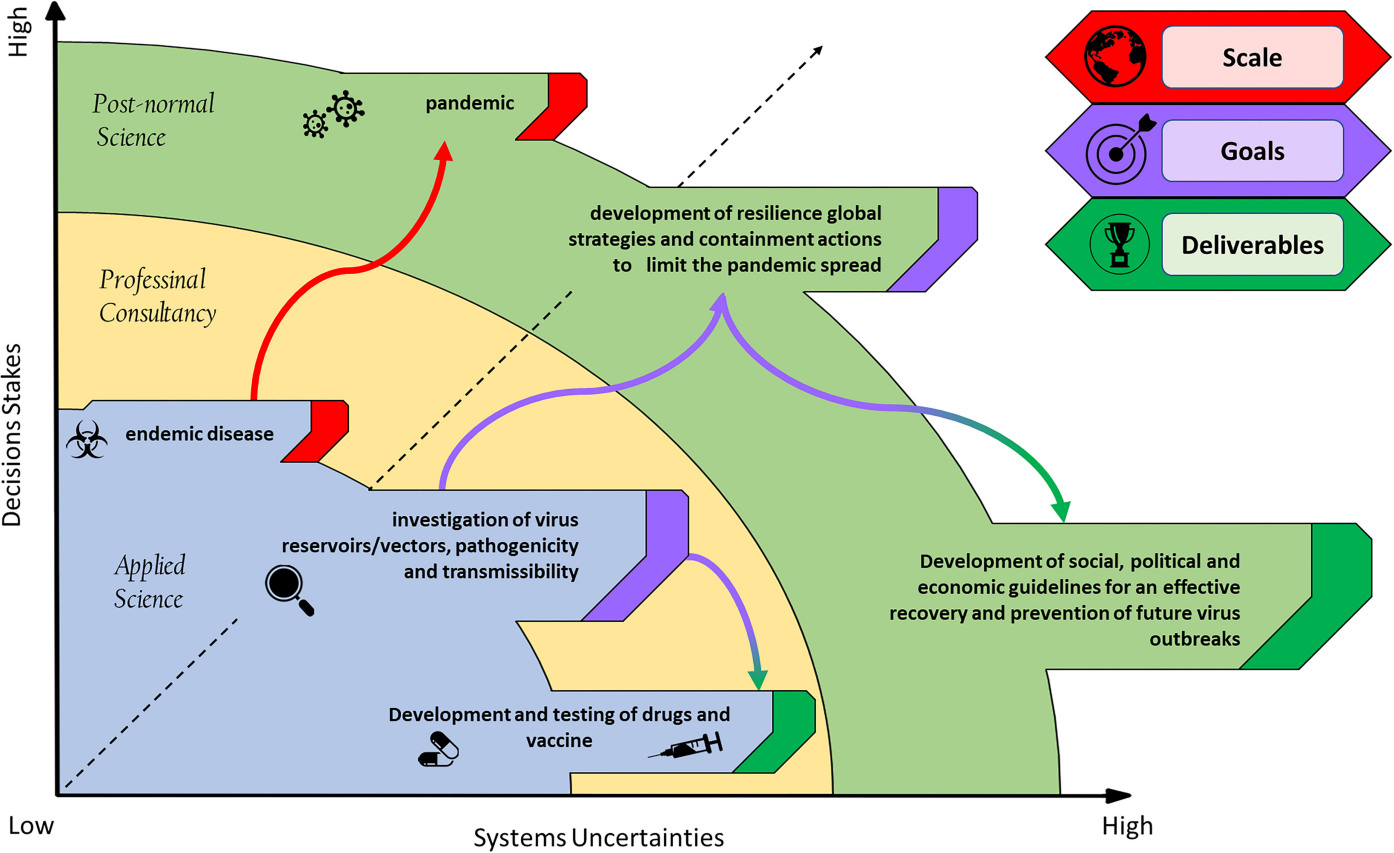
Schematization of a virus pandemic outbreak: scale, goals, and deliverables in a post-normal science context. The post-normal science diagram [taken from (7)] provides a framework to map systems uncertainty against decision stakes.

We believe that unlike the pandemics of the past, the factors triggering the current spread of COVID-19 outbreak, should be analyzed not only by scientists and politicians but also by societal stakeholders. Many European countries most afflicted with COVID-19 have started thinking ahead are now facing with the “phase two” of the COVID-19 situation by operating the recovery of industrial and social activities keeping, at the same time, the infection spread as low as possible. In such a context, the linear science, which analyzes those biological variables dealing with the pathogen and its infectivity to find possible solutions [e.g., a vaccine or a therapy (4)], clashes with the “Post-Normal Science” (PSN) approach, for which, societal values (e.g., the right to freedom, economic needs, and relational aspects) claim their importance (5). PNS is designed to deal with situations of uncertain facts, values in dispute, high stake and urgent decisions [(6); Figure 1]. PNS should be operated together with Responsible Research and Innovation (RRI) tools (https://www.rri-tools.eu/about-rri) that offer a broad set of strategies to address global challenges throughout the analysis of “real-world complexities' considering new scientific knowledge and technologies, but also the needs of different stakeholder categories. The contrast between these approaches to science is exerting some pressure on governments that even proposed solving the problem of COVID-19 with autonomous strategies or hypothesized to reach a sort of herd immunity by sacrificing an entire generation of older people (8, 9). This situation demonstrates the lack of organization by the modern society to address complex global issues.

Our contribution aims at supporting the adoption of a PNS response to COVID-19 pandemic, also considering the geopolitical and social aspects which caused the dramatic susceptibility to such infection. For this reason, we aimed at better addressing two main concepts regarding COVID-19 “effect” on cities and citizen-led community responses, to prevent future pandemic events.

## Pandemics Prevention

The first point to discuss is the unexpected permeability of cities to this virus and hypothesize how the spillover from wildlife of such a kind of pathogens can reach urban centers. This evaluation is of primary concern to better define suitable prevention strategies for limiting or blocking the current one and other future pandemic spreads. What characterizes COVID-19 contagion, is that it fully took advantage of globalization facilities, enabling rapid spread of the virus across the world.

Generally, accurate controls are performed on goods transported worldwide but in case of novel pathogens (e.g., the COVID-19) diagnostic options are very limited. Moreover, when human mobility is a major factor in the spread of infectious diseases, we should acknowledge that screening measures adopted at airports or customs should be implemented (10). Temperature screening alone may not be very effective as it may miss travelers incubating the disease or concealing fever during travel, or it may yield false positives (e.g., having fever of a different cause), therefore it should be accompanied by other health messages, questionnaires and data collection (11).

A different scenario occurs for the trading of food commodities for which strict regulations about quality and safety impose the adoption of analytical tools and innovation technologies to prevent the spread of foodborne pathogens or other contaminants (12, 13). In most cases, food quality evaluation is based on bioindicators, both at chemical and microbiological level (14, 15). Most diagnostic techniques allow to characterize the internal microbiome and virome (16–19). Considering COVID-19, what can be said with certainty is that such controls did not occur in the Wuhan market where the virus started its incredible global spread. This situation further remarks the concept that pandemics are strictly linked to insufficient or absent food safety assessment and disease prevention protocols (e.g., as happening every time Ebola Viruses outbreak in Central Africa). It is necessary to remark that the food regulations across countries vary and the quality standards of the same food items produced in different countries are not the same (20, 21). The current COVID-19 pandemic, perhaps more than others, highlights that it is necessary to align food security protocols on a global scale since country specific inadequacies may cause serious global consequences. Therefore, we believe that to prevent future pandemic outbreaks it is more and more important to consider issues arising from the establishment of supranational risk-governance systems. These typically operate in a framework of compromise between the local governance and local producers needs and the global safety for human health. Safety is a priority for all the stakeholders associated with the entire food supply-chain. Moreover, after the COVID-19 emergency, it is desirable that citizens enhance its awareness toward the topic of food control and the concept of food safety will acquire a stronger social meaning based on “shared values, beliefs and norms that affect human mindset and behavior” (22). Consumers' behavior and choices will help modify food supply-chains safety to prevent zoonoses and reduce other risks for humans.

Global citizenship is based on rights to be actively involved in debates, responsibility, shared decisions (following careful risk evaluation), and actions to control and implement shared strategies. With regard to COVID-19, we know that a bat species and/or the Malayan Pangolin have been found to be likely reservoir hosts for the virus; however, the definitive identity of any intermediate host that might have facilitated spillover to humans is still unknown (23, 24). Overall, the identification of the vector has a relatively important value. The central point is that the unceasing exploitation of wildlife and habitat has dramatically increased the risk of exposure to zoonotic diseases, as already and sadly demonstrated for example by HIV, Ebola and H5N1 (25). But how calculating the risk of these phenomena? Are stakeholders and citizens aware of the risks? These elements are fundamental for a PNS discussion that is necessary to drive the path of food safety.

It is time to realize that food safety cannot rely only on the production chain but potential risks to human health and the environment should be considered as well. The increasing advances in scientific and technological tools have now been adopted to assess such risks, thus opening a new era of “prediction” rather than “reaction” to reduce pathogen contamination and foodborne outbreaks (26). For example, the current trends in food safety research rely on the application of (i) genomic analyses for foodborne pathogen identification and traceability, (ii) Geographic Information Systems (GIS) to prevent and predict the spatial spread of pathogens outbreak, (iii) tools adapted from landscape ecology (species distribution and niche modeling) and Social Network Analysis for predicting patterns of disease outbreaks, as well as guidance for interventions, and (iv) meta-analysis tools to confer an overall summary of available study findings, providing generalizable estimates and generating strategic highlights to be used by policy-makers and decision makers (26).

**Figure 2:**
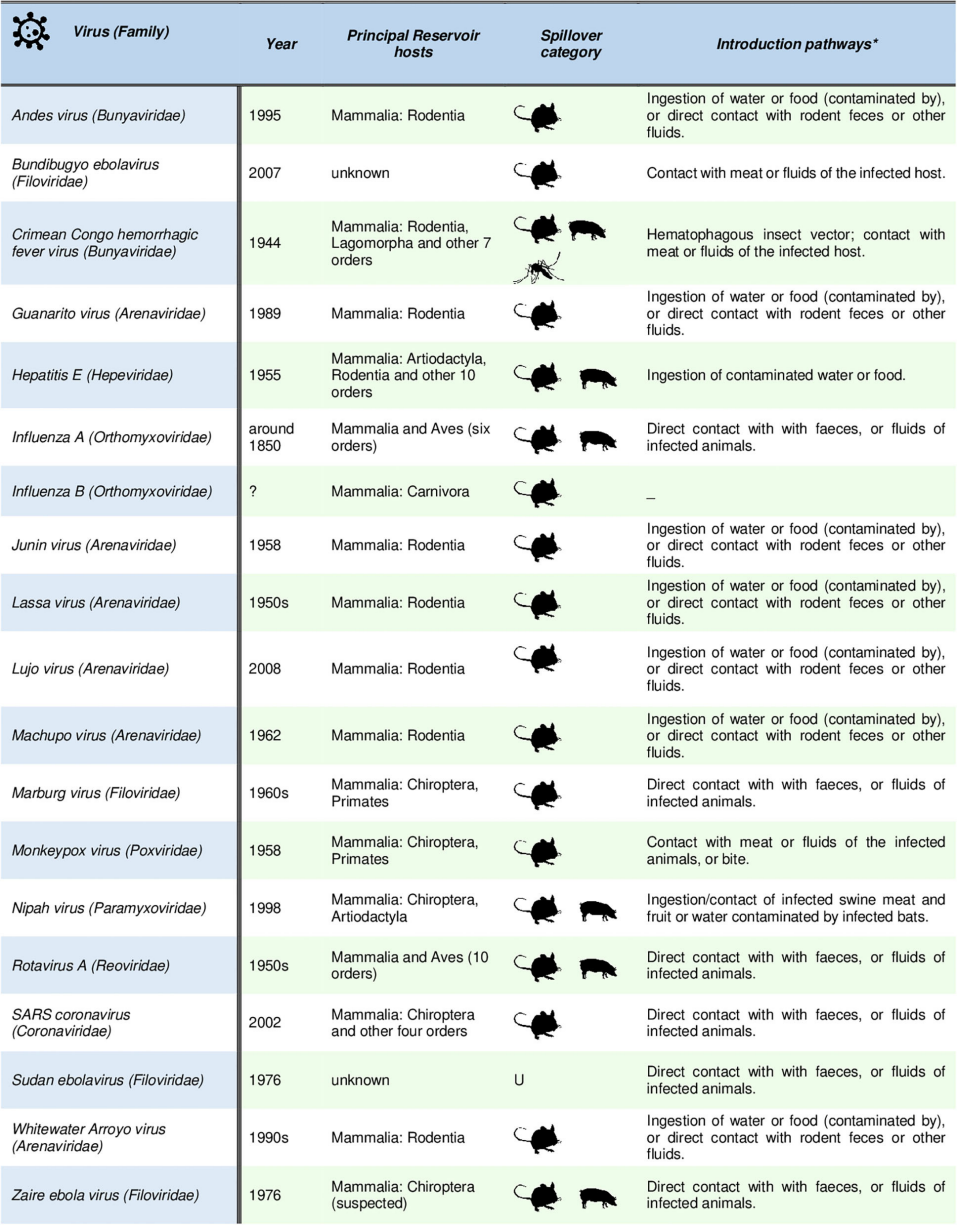
List of the principal pandemic and emerging zoonotic viruses showing human-to-human transmission after the spillover is occurred. Summarized host and spillover interface data are provided as in (29) (mouse: directly from wildlife; swine: directly from domestic animals; mosquito: transmission by vector involving wildlife or domestic host respectively; U: unknown). Detailed references for each listed virus are provided in (29).

Overall, risk prevention remains the key factor. The history of pandemics teaches us that almost all recent human pandemics and most of the emerging infectious diseases originated from animals (mainly in wildlife). It is known that species more resistant to human pressure are likely to become the new competent hosts of vector-borne diseases and then to become the most probable spillover agents toward human hosts (27, 28). Furthermore, we must remember that biodiversity perturbation and its trivialization is the main trigger of virus spillover events (29), as probably happened for COVID-19 (Figure 2).

Given these assumptions, the international food policies concerning food safety should consider biodiversity and ecological interventions to prevent zoonotic spillover events. This would be especially urgent in rural areas, where farming and livestocking often overlap with wildlife species ranges and it has been documented that livestock species usually act as intermediate hosts of spillover events (e.g., influenza A and SARS coronavirus, Figure 1). For this reason, it is also time to rethink urban areas by projecting proximity buffer zones to prevent direct contact between agricultural/zootechnic activities and natural habitats. Finally, the conservation of natural biodiversity and its related species interactions are essential conditions to reduce the risk of spillover events (27). On the whole, a cooperative work (RRI-driven) involving human-health agencies, agricultural authorities, farmers, and natural resource managing institutions, could be essential to promote the global ecological management to avoid the spread of a new putative pandemic “COVID-20” or other risky vector-borne pathogens that may adversely affect human health, the environment and economy.

## Citizen Fortification

Our second consideration regards the fragility of citizens, especially the weakest ones such as the elderly, and their sensitivity to diseases.

It is now clear that these social categories are the most susceptible to severe COVID-19 outcomes, particularly if they already suffer from multiple pathologies. Diabetes is the most common comorbidity observed in infected deceased patients in Italy, after hypertension (30). Furthermore, recent data showed a high prevalence of obesity (26%) and overweight (41%) in 928 Italian patients, median age 65 years, from 76 different Italian ICUs, confirming evidence available so far in the literature supporting impaired immune response to viral infections (31). Therefore, beyond the infectious capacity of this virus, it is important to focus on those elements of modern society which could increase citizens' vulnerability, including diet, lifestyle and environmental factors, strictly linked to morbidity and mortality for all Non-Communicable Diseases (NCDs) (32, 33). Although this concept is well-established, today, the global average consumption of healthy foods is substantially lower than the reference dietary intake, whereas overconsumption of highly processed, energy dense, and nutrient-poor foods is increasing (34).

The Mediterranean diet is considered by UNESCO as one of the “Intangible Cultural Heritage of Humanity” with multiple health benefits, including fortification of immune defenses. However, epidemiological data on COVID-19 would seem to contradict this belief since Mediterranean countries (e.g., Italy and Spain) have the highest number of confirmed COVID-19 cases in the world. Five years after the EXPO 2015, dedicated to the theme “Feeding the Planet, Energy for life,” the city of Milan, which hosted the event, is under siege by COVID-19 pandemic. Recent dietary changes within the Mediterranean basin, with a decreased consumption of plant foods, increased consumption of fast meals and junk food, and negative health consequences such as rise in obesity rates and in NCDs incidence (e.g., diabetes, cardiovascular diseases, and cancer) are partially responsible of this burden (35).

The modern-day change in food choices is the results of lifestyle standardization, enhanced technologies in food production and processing and limited time for culinary activities. This caused for example the progressive erosion of Mediterranean food cultures (36, 37). Moreover, environmental emergence, such as water scarcity in most Mediterranean countries and land wasting also has deleterious consequences on Mediterranean food production. Global climate changes have also produced the failure of several crops, fisheries, and livestock productions, and the declining of Mediterranean biodiversity and agrobiodiversity does not allow the selection of new varieties and resistant breeds. So, once again the erosion of the environment and biodiversity is closely connected to health risks (38). How to react to these social and environmental changes showing serious health consequences? The sustainability of the food supply chain is certainly essential (34). Kinnunen et al. showed that less than one-third of the world's population can meet their food demand within a 100-km radius (39). We should also change our view of food and diet which should no longer, or rather not only, considered an energy source but a reservoir of bioactive molecules beneficial to human health. Greater consumption of health-promoting foods and limited intake of unhealthier options are intrinsic to the eating habits of certain regional diets such as the Mediterranean diet (32). Healthy dietary patterns positively influence health and promotes the prevention of common non-communicable diseases (NCDs), strengthening host community defenses (32, 36). This concept assumes a particular importance since the over 65 years old citizens could be more at risk of being infected by COVID-19, not only for intrinsic conditions due to natural aging processes and comorbidities development, but also for inadequate nutritional status and related inadequate intake of macronutrients (e.g., proteins and healthy fatty acids, like omega-3), micronutrients (e.g., vitamins A, B6, B12, C, D, E, and folate), trace elements (e.g., zinc, iron, selenium, magnesium, and copper) and phytochemicals which are pillars in preventing many chronic degenerative diseases and supporting the immune system (40).

This would seem to be true even for younger patients with metabolic and cardiovascular diseases, showing severe Acute Respiratory Distress Syndrome (ARDS) caused by COVID-19 (41). These considerations are also well-known for past pandemics. A recent retrospective data analysis from the 1918 pandemic flu, showed that nutrition played a consistent role in the severity of the disease and was related to mortality also in younger age groups (42). More recently, chronic malnutrition has been correlated to high morbidity and mortality during the 2009 influenza pandemic (43). Similarly, malnourished children appear to be at increased risk for viral pneumonia (44).

In dietary recommendation, fat quality has to be addressed (45), since evidence shows the need to achieve a balance between dietary intake of omega-6 and omega-3 for optimal nutrition (46), especially in those subjects more vulnerable to malnutrition and “silent inflammation” which disposes to a greater propensity to viral infections (47, 48). Certainly, the presence of an unbalance between pro- and anti-inflammatory lipid mediators has been reported in literature (49) and it is important to remark that science has already given solutions (50) which are not adopted in new diagnostic policies for primary and secondary prevention.

Moreover, food supplements such as vitamins C and D might also be considered to help both innate and adaptive immune cells (51–54). Studies on human coronaviruses (HCoVs), including severe acute respiratory syndrome coronavirus (SARS-CoV), have highlighted that secondary metabolites of some plant species seem to inhibit virus proteins, cellular infection, and intracellular replication (55). Extracts of spontaneous plants such as root tubers from *Rheum officinale* Baill. (rhubarb), root tubers or vines from *Polygonum multiflorum* Thunb., (Polygonaceae) showed an inhibitory activity against the interaction of SARS-CoV S protein with ACE2 (56). Procyanidins and other secondary metabolites extracted from Cinnamomi cortex (*Cinnamomum cassia* J. Presl) can reduce the virus infection by interfering with endocytosis (57). The extract of *Cimicifuga* rhizoma, *Meliae cortex, Coptidis* rhizoma, *Phellodendron* cortex, and *Sophora subprostrata* radix showed an ability to inhibit of RNA-dependent RNA polymerase and/or proteases crucial for coronavirus RNA replication (58). Finally, the antiviral activity of licorice (*Glycyrrhiza glabra* L.), containing glycyrrhizin, inhibits replication, absorption and penetration of the SARS-CoV acting on the early steps of the replicative cycle (59). In this field, traditional Chinese medicine is working actively to identify dedicated compounds to specifically contrast COVID-19 infection (60). This practice relying on biodiversity and known as “bioprospecting,” may be a good strategy to find compounds having a positive effect on human health as well as to find new raw materials to produce novel and fortified foods for modern citizens.

## Pandemics and The Interaction With Environmental and Food Policy

**Figure 3:**
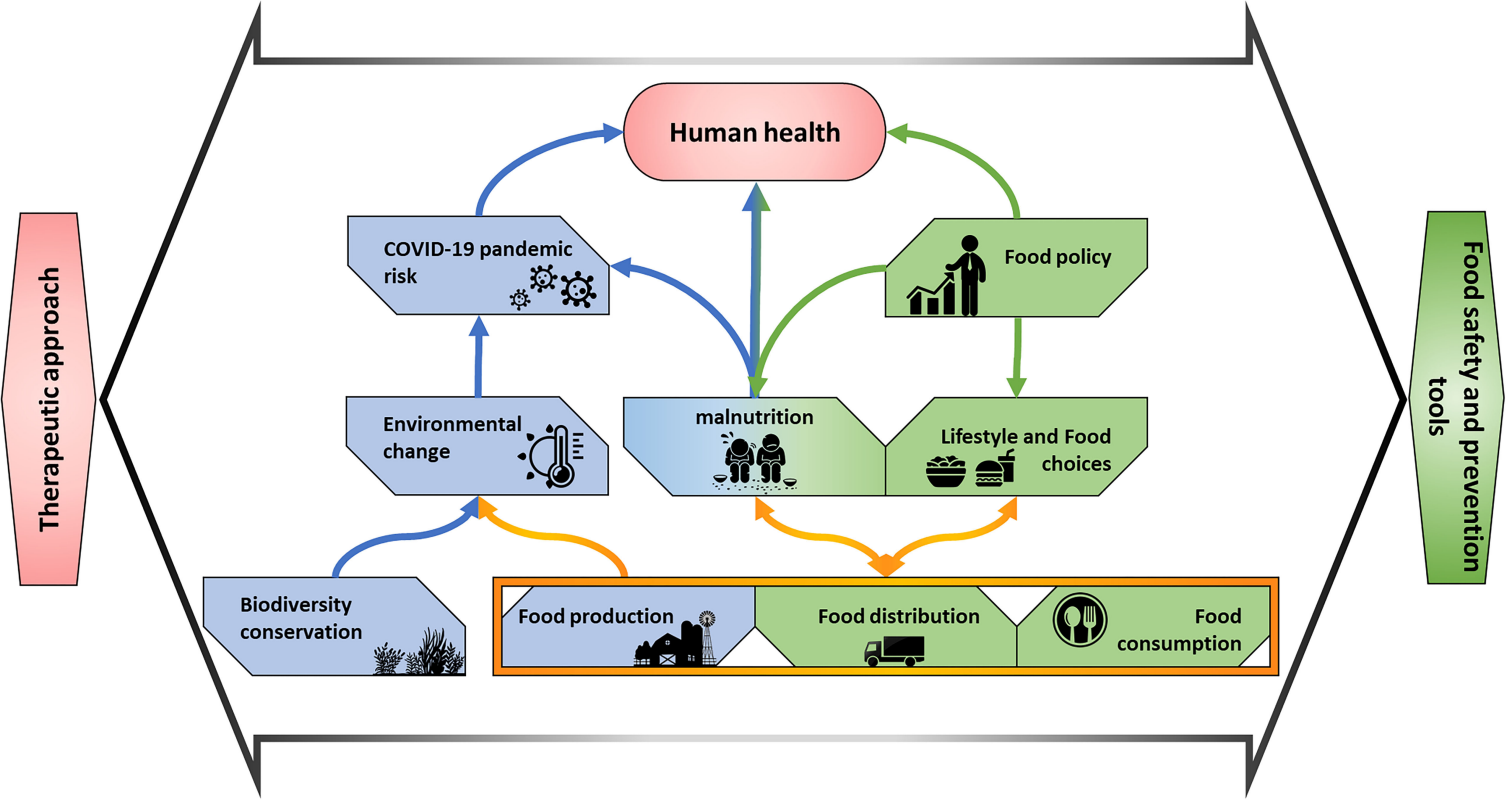
Network of interactions including food supply-chain elements and their effects (i) on environment and biodiversity that might lead to increased risk of spillover and (ii) on human health and wellbeing that affect infection risk prevention and therapy response.

Recently, Di Marco et al. (27) suggested that the risk of Emerging Infectious Diseases (EIDs) is a key aspect for developing suitable policy strategies at the global scale. Within this framework, the conservation of biodiversity and food production are two additional pillars that should be considered to prevent drastic environmental changes and the risks of zoonoses, and virus spread. In Figure 3 we aimed at further remarking this concept including additional factors strictly related to COVID-19 pandemic.

From a nutritional point of view, addressing subclinical micronutrient deficiencies is one of the first steps that must be considered to improve resistance to infectious diseases like COVID-19 (and other pathogens) (61). It is therefore recommended that micronutrient testing, such as vitamin D measurement, should be applied in the annual check up of selected individuals at high risk of deficiency (62).

A nutritional approach, ensuring a long-term sustainability, is essential to improve micronutrient status by increasing the availability and consumption of micronutrient-rich foods (63). Besides lifestyle changes, including diet has been shown to positively affect metabolic and cardiovascular diseases, which are the most frequent comorbidities associated to severe COVID-19 disease. The spread of inappropriate eating habits and inactivity in Western societies, particularly among the younger, “comfortably off” generations, has led to the development of chronic degenerative diseases defined as “comfort” diseases (64) early in life, leading to an increase in premature deaths for NCDs.

Food is readily available in developed countries but there is an evident split-up between scientific evidence, food choices, and dietary patterns of consumers. This, over time has favored the spread of the obesity epidemic and other diet-related diseases.

It is time to acknowledge that environmental factors exert a major influence on dietary behavior, primarily by facilitating meals consumption away from home and by minimizing time dedicated to meal preparation and consumption and secondly, making food of poor nutritional quality available on the market and appealing for appearance, taste and price. This burden is exerted by market rules that affect behavior and food choices with scarce public awareness of the potential negative impact on health. It is necessary that science, technology, education, legislation, and community policies combine to create the urban structures and environment required to encourage healthy lifestyle including dietary choices, not just for few, but for everyone (65).

More efforts must be addressed to reduce exposure to ambient air pollution, strongly associated with population density (66), promoting chronic inflammatory state and affecting resilience to infectious diseases not last COVID-19 (67).

Finally, Western medicine generally tends to identify pharmacological molecules that act on specific disease mechanisms; however, human body complexity and individual answers are sometimes underestimated. Thus, it could happen that infected patients die more due to comorbidities associated to infection with COVID-19 than for COVID-19 per se. The time has come to apply a systems biology approach where drugs, foods and lifestyle work in synergy to promote patient healing and prevent further infections, gaining a holistic approach to community health (68) to support the further personalization of health and social care (69).

For these reasons, in our scheme, we stressed the impact of environment and food system of therapeutic approaches.

This integrative framework demands both an increased attitude of sharing by the scientific and technical community as well as a social participation since we believe that, as previously anticipated, food safety and human health should be regarded more as a social issue.

## Conclusions

Our final suggestion is to start a frank and open scientific discussion on COVID-19 issues and future risks for new pandemic outbreaks, continuing the legacy of EXPO 2015, declared in the Milan Urban Food Policy Pact, signed by more than 200 cities in the world (65). In this document, the strategic role of the cities in developing sustainable food systems and promoting healthy diets is stated, yet acknowledging all the differences in their natural and policy endowments, including economic background and cultural innovation, managing vast public resources, infrastructure, investments, and expertise. These issues will be fundamental especially for those countries that are coming out of COVID-19 lockdown restrictions and where it is more expected that political and social contrast will emerge if different stakeholders needs and opinions will not be analyzed and considered for planning the recovery after the pandemic event.

We would like to undermine and integrate this overview with few take-home messages that arise from the teachings of this dramatic situation we are experiencing firsthand:

- Food safety is a global issue. The unsafety of local food markets, like the Wuhan's one, can exert severe and global impact.
- Smallholder food producers play a key role in feeding cities, by helping to maintain resilient, equitable, culturally appropriate food systems, and promote sustainable diets. In cities with a high percentage of elderly, local food production should be tailored for specific targets to maintain adequate nutritional status, including fortification of immune system. Similarly, in developing countries, smallholder farms should improve the production of local crops rich in macro and micronutrients to improve food security and health of local populations (70).
- Acknowledgment that urban and peri-urban agriculture may offer opportunities to protect and integrate biodiversity into urban landscapes and food systems, thereby contributing to synergies across food security, ecosystem services, and human wellbeing. This is very important to prevent the spillover of viruses but also to offer better efficacy of new drugs synthesized to fight future diseases.

A unified approach to nutritional screening and assessment is recommended guiding toward integrated panels of biomarkers to investigate the nutritional status and predict future health outcomes of the individual and moving from stratified to personalized to precision nutrition (71).

The whole scientific community should start sharing directions, social actions and policy advocacy recognizing that the health of people is closely connected to the health of biodiversity and ecosystems where they live. In this context, the “One health” approach (https://www.cdc.gov/onehealth/basics/index.html) represent a good starting point for successful public health interventions by fostering collaboration across all sectors, taking the right steps to promote health not for the few but for the whole planet.

## Data Availability Statement

The original contributions presented in the study are included in the article/supplementary materials, further inquiries can be directed to the corresponding author/s.

## Author Contributions

This opinion paper was conceived by ML, AG, and HC. All authors contributed to its execution and write up with ML leading on the manuscript structure, biodiversity-related aspects were contributed AG and MC, nutrition aspects by HC, botanic and phytochemistry aspects by MD, ES, EF, and LC, and medical aspects by MR and MB.

## Conflict of Interest

The authors declare that the research was conducted in the absence of any commercial or financial relationships that could be construed as a potential conflict of interest. The reviewer ML declared a past co-authorship with one of the author MR to the handling editor.
